# The Cystine/Glutamate Antiporter, System x_c_^–^, Contributes to Cortical Infarction After Moderate but Not Severe Focal Cerebral Ischemia in Mice

**DOI:** 10.3389/fncel.2022.821036

**Published:** 2022-05-09

**Authors:** Yan He, Sandra J. Hewett

**Affiliations:** Program in Neuroscience, Department of Biology, Syracuse University, Syracuse, NY, United States

**Keywords:** mouse, cystine/glutamate exchanger, photothrombosis, system x_c_^–^, permanent ischemia, transient ischemia

## Abstract

Understanding the mechanisms underlying ischemic brain injury is of importance to the goal of devising novel therapeutics for protection and/or recovery. Previous work in our laboratory and in others has shown that activation of cystine/glutamate antiporter, system x_c_^–^ (Sx_c_^–^), facilitates neuronal injury in several *in vitro* models of energy deprivation. However, studies on the contribution of this antiporter to ischemic brain damage *in vivo* are more limited. Since embolic or thrombotic transient or permanent occlusion of a cerebral blood vessel eventually leads to brain infarction in most stroke cases, we evaluated the contribution of Sx_c_^–^ to cerebral ischemic damage by comparing brain infarction between mice naturally null for SLC7a11 (SLC7a11^sut/sut^ mice) – the gene the encodes for the substrate specific light chain for system x_c_^–^ – with their wild type (SLC7a11^ + ⁣/ +^) littermates following photothrombotic ischemic stroke of the middle cerebral artery (PTI) and permanent middle cerebral artery occlusion (pMCAo) rendered by cauterization. In the PTI model, we found a time-dependent reduction in cerebral blood flow that reached 50% from baseline in both genotypes 47–48 h post-illumination. Despite this, a remarkable reduction in incidence and total infarct volume of SLC7a11^sut/sut^ mice was revealed 48 h following PTI as compared to SLC7a11^+/+^ mice. No difference in injury markers and/or infarct volume was measured between genotypes when occlusion of the MCA was permanent, however. Present data demonstrate a model-dependent differential role for Sx_c_^–^ in focal cerebral ischemic damage, further highlighting that ischemic severity activates heterogeneous biochemical events that lead to damage engendered by stroke.

## Introduction

It is well established that neuronal cell death in the acute phase of cerebral ischemia is caused by extracellular accumulation of the excitatory amino acid glutamate and consequent over-stimulation of postsynaptic glutamate receptors (Meldrum et al., 1985; Choi, 1988; Benveniste, 1991; Lipton and Rosenberg, 1994; Dirnagl et al., 1999). Surrounding this ischemic core is a region of reduced blood flow (10–50%) known as the penumbra. Evidence from both animal and human studies indicates that injury in this area continues to progress for hours and perhaps days following disruption of blood flow to the core (Heiss et al., 1992; Garcia et al., 1993; Du et al., 1996; Marchal et al., 1996; Baird et al., 1997; Schwamm et al., 1998). The mechanisms contributing to progression of injury in the penumbra are incompletely understood, although studies suggest that this may ensue from hypoxic spreading depression-like depolarizations that promote ongoing excitotoxicity (Somjen, 2001; Pietrobon and Moskowitz, 2014). Using a mixed cortical cell culture system, we previously found that astrocytic system x_c_^–^ (Sx_c_^–^)—a cystine/glutamate antiporter that exports Glu when importing cystine—contributed to glutamate-mediated excitotoxic neuronal death under simulated ischemic penumbral conditions (Fogal et al., 2007; Jackman et al., 2010, 2012). Others demonstrated that pharmacological block of Sx_c_^–^ reduced oxygen-glucose deprivation-induced neuronal currents (i.e., anoxic depolarizations) and cell death in slice and slice cultures, respectively (Soria et al., 2014), as well as in cortical cells (Hsieh et al., 2017). *In vivo*, a rapid increase in Sx_c_^–^ activity in rat brain after focal experimental cerebral ischemia induced by transient middle cerebral artery occlusion (tMCAo) was found (Soria et al., 2014) along with increased xCT protein levels localized to microglia/macrophages, neurons and astrocytes (Domercq et al., 2016; Hsieh et al., 2017). All together, these data support the idea that Sx_c_^–^ may play a deleterious role in brain damage that follows cerebral ischemia. Thus, in this study we determined the contribution of Sx_c_^–^ to neocortical infarction engendered by moderate or permanent focal ischemia, by comparing brain damage of mice wild type (*Slc7a11^+/+^*) or null (S*lc7a11*^sut/sut^**) for Sx_c_^–^ following photothrombotic ischemia (PTI) or permanent middle cerebral artery occlusion (pMCAo), respectively.

## Materials and Methods

### Animals and Animal Husbandry

This study was conducted in accordance with the National Institute of Health guidelines for the use and care of experimental animals as approved by the Institutional Animal Care and Use Committee. We utilized male mice on the C3H/HeSnJ background that have a naturally occurring deletion mutation in *Slc7a11*, the gene that encodes for the substrate-specific light-chain for Sx_c_^–^ (xCT) (Chintala et al., 2005). Experimental littermate mice—wild-type (Slc7a11^+/+^) or lacking system x_c_^–^ (Slc7a11^sut/sut^)—were derived from F1 heterozygous (Slc7a11^+/sut^) breeding units created by crossing Slc7a11^sut/sut^ C3H/HeSnJ male mice [Jackson Laboratories (JAX) Stock #001310] with Slc7a11^+/+^ C3H/HeSnJ female mice (JAX, Stock #000661). Mice were only used up to the F3 generation.

At weaning, genotyping was performed *via* PCR analysis of tail genomic DNA samples as described (Sears et al., 2019), after which mice were housed three to five per cage such that at least one mouse of each genotype was represented (pseudo-randomized design). Genotype was reconfirmed *via* PCR upon sacrifice. Mice were maintained in a controlled temperature environment operating on a 12 h light/dark cycle with standard mouse chow and water provided *ad libitum.* These breeding and housing strategies were utilized to control for environmental differences, genetic background influences, and genetic drift (Wolfer and Lipp, 2000; Wolfer et al., 2002).

### Ischemic Stroke Models

On each of the 5 days prior to a study, mice (23–30 g; 9–14 weeks) were physically held so that they would become accustomed to being touched. On the surgical day, mice were brought into the procedure room, weighed, and allowed to acclimatize for at least 1 h. Investigator was blind to mouse’s genotype at time of experimentation (i.e., surgery) and during all subsequent analyses. All mice were sacrificed 48 h after the induction of cerebral ischemia as described below. Supplementary Figure 1 provides a diagrammatic schematic depicting the timeline of each protocol. In both models, ischemic damage is largely restricted to the neocortex.

### Photothrombosis-Induced Ischemia

Cerebral ischemic damage was induced *via* photothrombosis *via* laser irradiation of the photosensitive compound Rose bengal (Boquillon et al., 1992; Ding et al., 2009). Mice, fully anesthetized with avertin (0.4 g/kg), were placed in a stereotaxic frame using tooth and ear bars. Using aseptic technique, the skull was exposed by making a 1.5–2 cm incision above the sagittal suture. A 5.5 mm × 5.0 mm rectangle metal sheet with a 1.5 mm diameter hole in the middle was gently attached to the right frontal-parietal area with glue (3M Vetbond ^®^). Freshly made Rose bengal (Abcam, Cambridge, United Kingdom) was then injected retro-orbitally (0.03 g/kg in saline) using a 28-gauge, 1/2 inch, 0.5 ml insulin needle and syringe. Three minutes after injection, the frame containing the mouse was mounted on an upright microscope platform and the exposed but otherwise intact skull was illuminated through the 1.5 mm diameter hole for 2 min using a 10x objective with a green light of bandwidth 540–580 nm by means of an X-cite 120Q light source to activate the dye to induce a thrombus. A heating pad was used to maintain mouse’s body temperature (36.5–37°C) during and following the procedure. Mortality with this procedure is very low with only one mouse of each genotype lost during surgery.

Ischemic damage was determined 48 h after surgery. Mice were perfused with cold PBS followed by 4% (v/v) paraformaldehyde (PFA). After perfusion, brains were removed and post-fixed in 4% PFA/PBS for 12 h and then placed in 20% (w/v) sucrose for an additional 12–24 h (4°C). Tissue was then embedded in Tissue Tec OCT and solidified in liquid nitrogen. Brain sections (20 μm) cut serially through the rostro-caudal extent of each brain (−2.0 to −3.6 relative to bregma) (Microm HM-550 cryostat, Thermo Fisher Scientific) were stained with 0.5% thionin as described in detail (Claycomb et al., 2011). Images were acquired by a DP73 digital color camera (Digital Video Camera Co.) mounted on an Olympus IX50 inverted microscope controlled by CellSens Standard software (Olympus, Center Valley, PA). Infarct area (A) was directly measured using the free hand tool of ImageJ by tracing the area of the ipsilateral cortex that lacked thionin stain. The lesion area, identified by absence of thionin staining, was quantified using NIH Image J at four levels spanning from −2.0 to −3.6 from bregma by two individuals blind to genotype. Area measurements were converted to volume using Cavalieri’s principle: V = Ai × D + Aii × D + Aiii × D + Aiv with V = total infarct volume (mm^3^) and Ai = mean infarct area of each section derived from two measurements and D represents the distance between 2 sections (Shih et al., 2003).

Laser Speckle Contrast Analysis was used to measure microvascular cerebral blood flow in the right MCA territory where the Rose Bengal was activated [PSI HR real time laser speckle perfusion imager; Pericam (Las Vegas, NV)] in a separate cohort of mice (see Supplementary Figure 2 for representative video). Briefly, after making the incision and exposing the skull, mouse was moved to the imager to access the baseline of blood flow for 30 sec per manufacturer’s instruction. Then a 5.5 mm × 5.0 mm rectangle metal sheet with a 1.5 mm diameter hole in the middle was gently attached to the right frontal-parietal area with glue (3M Vetbond ^®^), followed by the retro-orbital injection and laser activation of Rose Bengal as described above. After the activation, the metal sheet was gently removed, and mouse was immediately moved to the imager to access the real time blood flow for 30 sec (time zero). The incision was sealed lightly by glue, after which the mouse was put back into their cage containing a heating-pad and allowed to recover. Mice were re-anesthetized, the incision gently re-opened and additional blood flow measurements taken over the same region at 4–6, 24, and 47–48 h post-illumination. Data at each time point was normalized to baseline for each mouse and expressed as mean percentage ± S.D.

### Permanent Middle Cerebral Artery Occlusion

Permanent cerebral cortical ischemia was induced in avertin-anesthetized male mice by ligating the right common carotid artery (CCA) and cauterizing the right middle cerebral artery (MCA) distal to the striatal branch as described in Piao et al. (2009) and Cui et al. (2015). The mouse’s body temperature was maintained at 36.5–37°C during the surgery and during recovery *via* heat pad. With respect to mortality, we lost one Slc7a11^+/+^ and two Slc7a11^sut/sut^ mice 1 day after surgery. Those surviving were sacrificed 48hr later and brains directly sectioned into 1 mm coronal sections. The 4th, 6th, 8th, and 10th sections were stained with 2% 2,3,5-triphenyl-tetrazolium chloride (TTC; Sigma-Aldrich, St. Louis, MO) for 30 min at 37°C in the dark. Slices were then carefully transferred into 10% formalin for 24 h. Images (300 dpi) were captured by scanning (Epson Perfection 3170). The non-injury areas of both contralateral and ipsilateral hemispheres—denoted by red staining—were measured using NIH ImageJ for all sections by two experimenters blind to the genotype of the mice and infarct area and volume determined. Infarct area (A) was determined by subtracting the non-injured ipsilateral area from the area of the contralateral hemisphere. Total infarct volume (mm3) was calculated using the following equation: V = Ai × 2 + Ai × 2 + Aii × 2 + Aiii x 2 + Aiv with V = total infarct volume and Ai = infarct area of each section with 2 representing the distance between slices (i.e., 2 mm) (Shih et al., 2003).

To measure spectrin breakdown products, shown previously to correlate nicely with final infarct volume (Davoli et al., 2002), contralateral and ipsilateral sides of the 5th, 7th, and 9th sections were separately pooled and homogenized in RIPA buffer containing: 0.5% Sodium deoxycholate, 25 mM Tris, 150 mM NaCl, 0.1% SDS, 1% Triton X-100, 5 mM iodoacetamide, 5 mM EDTA, 5 mM EGTA, and 1 X Complete Protease Inhibitor (Roche). Cellular debris was removed by centrifugation (12,000 g; 20 min; 4°C). Supernatants were collected and 15 μg (BCA assay; Pierce; Rockford, IL) of protein was separated by 10% SDS-PAGE under reducing conditions, followed by electrophoretic transfer to PVDF membrane (Bio-Rad; Hercules, CA). Membranes were blocked (Odyssey ^®^ blocking buffer; at 25°C for 1 h) and then probed (4°, overnight) with an anti-α Fodrin monoclonal antibody (AA6, 100 ng/ml; Enzo) and a mouse monoclonal antibody directed against β-actin to correct for protein loading (650 ng/ml; Sigma). Species-specific secondary antibodies labeled with spectrally distinct IRDye ^®^ fluorescent dyes (LI-COR Biosciences; Lincoln, NE) were used to detect primary antibodies (1 h at 25°C). Results were recorded on LI-COR ODYSSEY ^®^ Fc Imaging system (LI-COR Biosciences) and protein levels quantified using Image Studio 3.1 (LI-COR Biosciences; Lincoln, NE). Results were obtained by normalizing fodrin protein levels to their respective β-actin levels and expressed as mean fold change over the corresponding contralateral hemisphere (set to 1).

#### Statistical Analysis

All statistical analyses and graphics were compiled using GraphPad Prism (Version 6.0.3, GraphPad Software, Inc. or higher; Graphpad Software, Inc., La Jolla, CA). Cerebral blood flow (CBF) were analyzed using a mixed effects model with the Geisser-Greenhouse correction for normality followed by Šídák’s multiple comparisons test. αII-spectrin breakdown products (SBDPs) where analyzed using two-way ANOVA of log transformed data [y = log(y)] followed by Šídák’s multiple comparisons test. Infarct volumes were analyzed *via* two-tailed Mann Whitney *U*-test. Exact or adjusted *p*-values are included in text, in a table and/or described in figure legends as appropriate.

## Results

**FIGURE 1:**
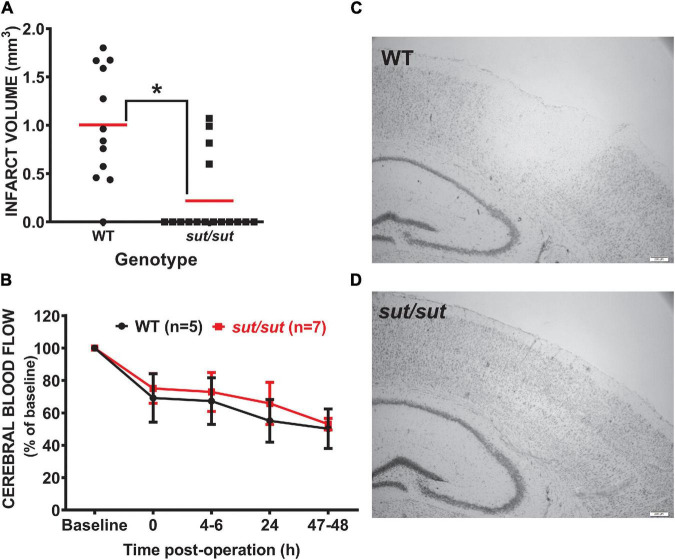
Comparison of infarct incidence and size between WT and Sx_c_^–^ null mice following photothrombotic-induced ischemia. **(A)** Forty-eight h after PTI, brains of sut/sut (*n* = 16) mice and their WT (*n* = 13) littermates were removed. Coronal sections (20 μm) were cut along the rostral-caudal axis (−2.0 to −3.6 relative to bregma) and stained with thionin, with areas lacking thionin staining represent ischemic damage. Infarct volume was determined as described in Methods. Each data point (black dot: WT; black square: sut/sut) represents total infarct volume in mm^3^ from an individual mouse, whereas the horizontal red line represents the mean **(B)** Depicts rCBF as determined *via* laser speckle contrast imaging represented as mean fold change ± *SD* from baseline (set to 100) taken immediately prior to illumination and at intervals following illumination as described in methods. **(C)** Representative thionin staining of brain tissue of Slc7a11^+/+^ (WT) and **(D)** Slc7a11^sut/sut^ (sut/sut) mice. A significant difference in infarct volume (*p = 0.0007) was determined by two-tailed Mann Whitney *U*-test. Infarct incidence −defined as the number of mice with any size infarct divided by the total number of mice—also differed significantly between genotypes as assessed by Fisher’s exact test (*p* = 0.005).

Anatomical damage due to PTI was greatly reduced in mice lacking Sx_c_^–^ with overall infarct volume being significantly smaller (two-tailed Mann Whitney *U*-test; *p* = 0.0007) in SLC7a11^sut/sut^ as compared to SLC7a11^+/+^ littermate controls (Figures 1A,C,D). This change was driven mostly by a reduction in the overall incidence of damage evidenced by the fact that 92% of SLC7a11^+/+^ mice (12/13) had quantifiable cortical infarction as compared to just 25% (4/16) of SLC7a11^sut/sut^ mice (Figure 1A; Fishers exact test, *p* = 0.005). Differing amounts of occlusion could not account for the dissimilarity in PTI-mediated damage as similar decreases in rCBF starting at ≈25–30% (69.2 ± 14.9 vs. 75.1 ± 9.1 of baseline levels for SLC7a11^+/+^ vs. SLC7a11^sut/sut^, respectively) measured directly after laser illumination progressing to ≈50% reduction of pre-illumination levels (50.2 ± 12.2 vs. 53.0 ± 3.6 for SLC7a11^+/+^ vs. SLC7a11^sut/sut,^ respectively) by the time of sacrifice occurred irrespective of the mouse’s genotype (Figure 1B). Mixed effect model analysis shows a significant effect of time (*p* < 0.0001) with *post hoc* analyses revealing a significant reduction from baseline in SLC7a11^+/+^ mice occurring at 24 and 48 h, respectively. In contrast, significant changes from baseline occurred at all-time points post-illumination in SLC7a11^sut/sut^ mice. Pertinently, no significant between genotype differences was found at any time post-illumination (Table 1).

**TABLE 1:** Statistical analysis of rCBF following PTI (Figure 1B).

Blood flow	SLC7a11^+/+^	SLC7a11^sut/sut^	+/+ vs. sut/sut
			
Post-illumination time (h)	*P*-value (change from baseline)	*p*-value (change from baseline)	*p*-value (between group difference)
0	0.096	0.004	0.956
4–6	0.069	0.023	0.970
24	0.016	0.005	0.666
47–48	0.008	<0.0001	0.994

**FIGURE 2:**
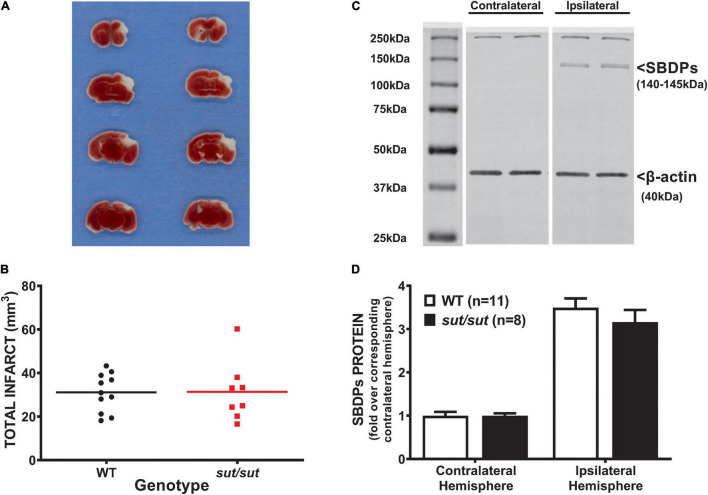
Comparison of pMCAo-induced brain damage between system x_c_^–^ null (*sut/sut*) mice and their wild type (WT) littermates. Forty-eight hr after pMCAo, brains were harvested from *sut/sut* (*n* = 8) mice and their WT (*n* = 11) littermates and cut in 1mm sections along the rostral-caudal axis. Sections 4, 6, 8, and 10 were used to quantify infarct volume, while the 5th, 7th and, 9th sections were used for immunoblot analysis of SBDPs, each as described in methods. **(A)** Representative TTC staining of coronal brain sections from WT (left panel) and *sut/sut* littermates (right panel). Lack of TTC staining delineates infarct. **(B)** Each data point (black dot: WT; red square: *sut/sut*) represents total infarct volume in mm^3^ from an individual mouse, whereas the horizontal line represents the mean of all mice. **(C)** Representative blot of SBDPs **(D)** SBDPs protein levels, normalized to their corresponding β-actin, are expressed as mean fold change + SEM over the corresponding contralateral hemisphere (set to 1).

In contrast to what we found with PTI, a well demarcated infarct was visible 48 hr following pMCAo in all mice regardless of genotype (Figure 2A). Analysis of infarct volume showed no significant difference in infarct size between SLC7a11^+/+^ and SLC7a11^sut/sut^ mice (two tailed Mann Whitney *U*-test, *p* > 0.999) (Figure 2B). The levels of αII spectrin breakdown products, measured as an additional index of ischemic injury, also did not differ between genotypes (Figures 2C,D; two-way ANOVA; *p* = 0.925).

## Discussion

Occlusion of a major blood vessel, most notably the middle cerebral artery (Olsen et al., 1985), leads to cerebral ischemia in the vast majority of stroke cases. The pace of brain circuitry loss in humans from a typical large vessel occlusion is estimated to be a staggering 120 million neurons and 830 billion synapses per hour (Saver, 2006). Given that > 750,000 persons in the US will have a stroke this year, it is imperative that more therapies be made available, which necessitates our need to understand more fully the mechanisms underlying ischemic brain damage. Previous PET imaging studies demonstrated increased Sx_c_^–^ activity in rat brain *in vivo* following transient focal ischemia produced *via* intraluminal occlusion of MCA followed by reperfusion (Soria et al., 2014; Domercq et al., 2016). The models chosen in this study to assess the role of Sx_c_^–^ to ischemic damage directly mimic different clinical conditions. Photosensitization of intravascular Rose Bengal by laser illumination (Watson et al., 1985; Pevsner et al., 2001) at the territory of the MCA induces platelet aggregation, vascular thrombosis and, as we demonstrated herein, incomplete blood flow reduction, while pMCAo *via* direct electrocoagulation mimics severe ischemic stroke without vessel recanalization (Sommer, 2017). Present data demonstrate that activity of Sx_c_^–^ promotes cortical infarction after moderate (PTI) but not severe (pMCAo) focal cerebral ischemia in mice.

The mouse utilized in this study was first identified by its subtle gray coat pigmentation that sequencing and subsequent experimentation revealed resulted from a large deletion mutation in Exon 12 of the *Slc7a11* gene (Chintala et al., 2005), which encodes the substrate specific light chain (xCT) for the cystine/glutamate exchanger, system x_c_^–^. Importantly, we and others (Swank et al., 1996) have found that on a non-agouti background the reduction in pigmentation arising from the sut mutation becomes nearly imperceptible when the mice are adults making homozygotes difficult to distinguish from wild-type littermate controls; hence our ability to remain blind to genotype during experimentation. Additionally, despite the identification of an alternative transcript *via* 3’RACE, northern blot analysis reveals no xCT mRNA in brain of Slc7a11^sut/sut^ mice (Chintala et al., 2005). This is likely due to the fact that loss of the 3’UTR *via* this deletion leads to absence of polyadenylation, which is necessary for mRNA stability and translation (Muhlemann and Jensen, 2012; Lykke-Andersen and Bennett, 2014). Additionally, no xCT protein in brain was found *via* Western Blot analysis (McCullagh and Featherstone, 2014).

We initially considered that changes in clotting −either initial aggregation or more pronounced lysis—might explain the results in the PTI model given a reduction in collagen-mediated platelet aggregation *ex vivo* has been reported in SLC7a11^sut/sut^ mice (Swank et al., 1996). However, our data showing a sustained reduction in CBF over the 48 hr period suggests this is not the case. This may not be too surprising given the complexity of factors/mechanisms known to facilitate platelet aggregation *in vivo* (Rumbaut and Thiagarajan, 2010). Given this, we conclude that the neocortical tissue of mice is remarkably less vulnerable to death when Sx_c_^–^ function is absent under conditions where blood flow is moderately disrupted (Figure 1).

Our PTI results are somewhat in keeping with those of Hsieh and colleagues, who demonstrated a reduction in infarct volume in genetically manipulated mice lacking Sx_c_^–^ (xCT^–/–^) when subjected to cerebral ischemia followed by reperfusion (Hsieh et al., 2017). Both of these findings might seem surprising given the importance of Sx_c_^–^ to the production of GSH, at least, *in vitro*, where growth of xCT-deficient cells is dependent on the addition of a reducing agent (Bannai and Tateishi, 1986; Chintala et al., 2005; Shih et al., 2006; Jackman et al., 2010). However, xCT^–/–^ and Slc7a11^sut/sut^ mice have normal brain GSH levels (De Bundel et al., 2011; Sears et al., 2019), at least under basal conditions, suggesting that other cyste(i)ne transporter systems compensate for loss *in vivo* (Sosnoski et al., 2020). We cannot discount that there might be GSH dysregulation following ischemic stress. However, if so, the impact would appear minimal as evidenced by a reduction in damage following PTI and a lack of injury enhancement after pMCAo.

Interestingly, following tMCAo, xCT^–/–^ mice had appreciably less extracellular glutamate levels in the ischemic cortex at early (2–5 hr) and late time points (1–3 days), indicating a role for Sx_c_^–^ as a source of extracellular glutamate post-ischemia (Hsieh et al., 2017). Relevant to this, we demonstrated increased astrocytic system x_c_^–^ levels and activity contributes to enhanced extracellular Glu levels, which precipitates excitotoxic neuronal cell death in an *in vitro* model of the ischemic penumbra (Fogal et al., 2007; Jackman et al., 2010). Together, these results help explain the intriguing observation made by Obrenovitch that neither vesicle exocytosis or reversed Glu uptake could account for all the cerebral ischemic extracellular Glu levels measured *in vivo* (Obrenovitch, 1996). Together, these results extend the current, perhaps oversimplified, concept of excitotoxicity that has heretofore dominated current thinking to include Sx_c_^–^. Given the lack of specificity of the commercial antibodies directed against xCT (Van Liefferinge et al., 2016), we did not explore its cell-type expression following ischemia in our models. However, others have demonstrated increased immunofluorescence for xCT in microglia/macrophages at 3 and 7 days post-ischemia and in astrocytes up to 28 days post-ischemia (Domercq et al., 2016) while Hsieh and colleagues report a post-ischemic increase in expression in both astrocytes and neurons (Hsieh et al., 2017).

Despite the evidence demonstrating the contribution of Sx_c_^–^ to ischemic damage when blood flow is moderately (this study) or transiently (Hsieh et al., 2017) reduced, a very different picture emerged when the MCA was permanently occluded. Finding no difference in infarct volume or other markers of injury (Figure 2), we can only conclude that other biochemical mechanisms clearly predominate when blood flow disruption is severe. This is perhaps not surprising as many potential therapeutics have been described in the literature that show potential promise in transient but not permanent ischemia models and the idea has been put forth that salvageable tissue exits only in the ischemic penumbra (for review see Moskowitz et al., 2010).

Studies in genetically modified mice are useful for potential target identification. However, in the interest of therapeutic development, it is important to note that in keeping with the observations described above, pharmacological inhibition of Sx_c_^–^ reduced ischemia-induced inflammation (Domercq et al., 2016) and infarct volume (Hsieh et al., 2017) subsequent to transient ischemia in rats, but not following permanent ischemia in mouse (our unpublished observations). While it is clear that additional studies are needed to determine the relevance of our and other findings to human stroke, it is intriguing to speculate that should strategies to mitigate Sx_c_^–^ activity ever be employed in a clinical setting, it may only be beneficial to patients whose vessel recanalizes leading to revascularization or in patients with penumbra where blood flow is mildly to moderately interrupted (10–50% of baseline).

## Data Availability Statement

The raw data supporting the conclusions of this article will be made available by the authors, without undue reservation.

## Ethics Statement

The animal study was reviewed and approved by the Syracuse University’s Institutional Animal Care and Use Committee.

## Author Contributions

YH and SH: conceptualization, methodology, funding acquisition, writing of original draft, reviewing and editing, and statistical analysis. YH: investigation. SH: resources and supervision. Both authors contributed to the article and approved the submitted version.

## Conflict of Interest

The authors declare that the research was conducted in the absence of any commercial or financial relationships that could be construed as a potential conflict of interest.

## Publisher’s Note

All claims expressed in this article are solely those of the authors and do not necessarily represent those of their affiliated organizations, or those of the publisher, the editors and the reviewers. Any product that may be evaluated in this article, or claim that may be made by its manufacturer, is not guaranteed or endorsed by the publisher.

## References

[B1] Baird A. E. Benfield A. Schlaug G. Siewert B. Lovblad K. O. Edelman R. R. (1997). Enlargement of human cerebral ischemic lesion volumes measured by diffusion-weighted magnetic resonance imaging. *Ann. Neurol.* 41 581–589. 10.1002/ana.410410506 9153519

[B2] Bannai S. Tateishi N. (1986). Role of membrane transport in metabolism and function of glutathione in mammals. *J. Membr. Biol.* 89 1–8. 10.1007/BF01870891 2870192

[B3] Benveniste H. (1991). The excitotoxin hypothesis in relation to cerebral ischemia. *Cerebrovasc. Brain Metab. Rev.* 3 213–245. 1931486

[B4] Boquillon M. Boquillon J. P. Bralet J. (1992). Photochemically induced, graded cerebral infarction in the mouse by laser irradiation evolution of brain edema. *J. Pharmacol. Toxicol. Methods* 27 1–6. 10.1016/1056-8719(92)90013-q 1581608

[B5] Chintala S. Li W. Lamoreux M. L. Ito S. Wakamatsu K. Sviderskaya E. V. (2005). Slc7a11 gene controls production of pheomelanin pigment and proliferation of cultured cells. *Proc. Natl. Acad. Sci. U S A* 102 10964–10969. 10.1073/pnas.0502856102 16037214 PMC1178012

[B6] Choi D. W. (1988). Glutamate neurotoxicity and diseases of the nervous system. *Neuron* 1 623–634. 10.1016/0896-6273(88)90162-6 2908446

[B7] Claycomb R. J. Hewett S. J. Hewett J. A. (2011). Prophylactic, prandial rofecoxib treatment lacks efficacy against acute PTZ-induced seizure generation and kindling acquisition. *Epilepsia* 52 273–283. 10.1111/j.1528-1167.2010.02889.x 21219314 PMC4445939

[B8] Cui L. Duchamp N. S. Boston D. J. Ren X. Zhang X. Hu H. (2015). NF-kappaB is involved in brain repair by stem cell factor and granulocyte-colony stimulating factor in chronic stroke. *Exp. Neurol.* 263 17–27. 10.1016/j.expneurol.2014.08.026 25281484

[B9] Davoli M. A. Fourtounis J. Tam J. Xanthoudakis S. Nicholson D. Robertson G. S. (2002). Immunohistochemical and biochemical assessment of caspase-3 activation and DNA fragmentation following transient focal ischemia in the rat. *Neuroscience* 115 125–136. 10.1016/s0306-4522(02)00376-7 12401327

[B10] De Bundel D. Schallier A. Loyens E. Fernando R. Miyashita H. Van Liefferinge J. (2011). Loss of system x_c_^–^ does not induce oxidative stress but decreases extracellular glutamate in hippocampus and influences spatial working memory and limbic seizure susceptibility. *J. Neurosci*. 31, 5792–5803. 10.1523/jneurosci.5465-10.2011 21490221 PMC6622811

[B11] Ding S. Wang T. Cui W. Haydon P. G. (2009). Photothrombosis ischemia stimulates a sustained astrocytic Ca2+ signaling in vivo. *Glia* 57 767–776. 10.1002/glia.20804 18985731 PMC2697167

[B12] Dirnagl U. Iadecola C. Moskowitz M. A. (1999). Pathobiology of ischaemic stroke: an integrated view. *Trends Neurosci.* 22 391–397. 10.1016/s0166-2236(99)01401-0 10441299

[B13] Domercq M. Szczupak B. Gejo J. Gomez-Vallejo V. Padro D. Gona K. B. (2016). PET Imaging with [(18)F]FSPG Evidences the Role of System xc(-) on Brain Inflammation Following Cerebral Ischemia in Rats. *Theranostics* 6 1753–1767. 10.7150/thno.15616 27570548 PMC4997234

[B14] Du C. Hu R. Csernansky C. A. Hsu C. Y. Choi D. W. (1996). Very delayed infarction after mild focal cerebral ischemia: a role for apoptosis? *J. Cereb. Blood Flow Metab.* 16 195–201. 10.1097/00004647-199603000-00003 8594050

[B15] Fogal B. Li J. Lobner D. McCullough L. D. Hewett S. J. (2007). System x(c)- activity and astrocytes are necessary for interleukin-1beta-mediated hypoxic neuronal injury. *J. Neurosci.* 27 10094–10105. 10.1523/JNEUROSCI.2459-07.2007 17881516 PMC6672668

[B16] Garcia J. H. Yoshida Y. Chen H. Li Y. Zhang Z. G. Lian J. (1993). Progression from ischemic injury to infarct following middle cerebral artery occlusion in the rat. *Am. J. Pathol.* 142 623–635. 8434652 PMC1886726

[B17] Heiss W. D. Huber M. Fink G. R. Herholz K. Pietrzyk U. Wagner R. (1992). Progressive derangement of periinfarct viable tissue in ischemic stroke. *J. Cereb. Blood Flow Metab.* 12 193–203. 10.1038/jcbfm.1992.29 1548292

[B18] Hsieh C. H. Lin Y. J. Chen W. L. Huang Y. C. Chang C. W. Cheng F. C. (2017). HIF-1alpha triggers long-lasting glutamate excitotoxicity via system xc(-) in cerebral ischaemia-reperfusion. *J. Pathol.* 241 337–349. 10.1002/path.4838 27801527

[B19] Jackman N. A. Melchior S. E. Hewett J. A. Hewett S. J. (2012). Non-cell autonomous influence of the astrocyte system xc- on hypoglycaemic neuronal cell death. *ASN Neuro.* 4:e00074. 10.1042/AN20110030 22220511 PMC3275339

[B21] Jackman N. A. Uliasz T. F. Hewett J. A. Hewett S. J. (2010). Regulation of system x(c)(-)activity and expression in astrocytes by interleukin-1beta: implications for hypoxic neuronal injury. *Glia* 58 1806–1815. 10.1002/glia.21050 20645408 PMC4451603

[B22] Lipton S. A. Rosenberg P. A. (1994). Excitatory amino acids as a final common pathway for neurologic disorders [see comments]. *N. Engl. J. Med.* 330 613–622. 10.1056/nejm199403033300907 7905600

[B23] Lykke-Andersen J. Bennett E. J. (2014). Protecting the proteome: eukaryotic cotranslational quality control pathways. *J. Cell Biol.* 204 467–476. 10.1083/jcb.201311103 24535822 PMC3926952

[B24] Marchal G. Beaudouin V. Rioux P. de la Sayette V. Le Doze F. Viader F. (1996). Prolonged persistence of substantial volumes of potentially viable brain tissue after stroke: a correlative PET-CT study with voxel-based data analysis. *Stroke* 27 599–606. 10.1161/01.str.27.4.599 8614914

[B25] McCullagh E. A. Featherstone D. E. (2014). Behavioral characterization of system xc- mutant mice. *Behav. Brain Res.* 265 1–11. 10.1016/j.bbr.2014.02.010 24548853

[B26] Meldrum B. Evans M. Griffiths T. Simon R. (1985). Ischaemic brain damage: the role of excitatory activity and of calcium entry. *Br. J. Anaesth.* 57 44–46. 10.1093/bja/57.1.44 3966963

[B27] Moskowitz M. A. Lo E. H. Iadecola C. (2010). The science of stroke: mechanisms in search of treatments. *Neuron* 67 181–198. 10.1016/j.neuron.2010.07.002 20670828 PMC2957363

[B28] Muhlemann O. Jensen T. H. (2012). mRNP quality control goes regulatory. *Trends Genet.* 28 70–77. 10.1016/j.tig.2011.11.001 22154474

[B29] Obrenovitch T. P. (1996). Origins of glutamate release in ischaemia. *Acta Neurochir. Suppl.* 66 50–55. 10.1007/978-3-7091-9465-2_9 8780797

[B30] Olsen T. S. Skriver E. B. Herning M. (1985). Cause of cerebral infarction in the carotid territory. Its relation to the size and the location of the infarct and to the underlying vascular lesion. *Stroke* 16 459–466. 10.1161/01.str.16.3.459 4002261

[B31] Pevsner P. H. Eichenbaum J. W. Miller D. C. Pivawer G. Eichenbaum K. D. Stern A. (2001). A photothrombotic model of small early ischemic infarcts in the rat brain with histologic and MRI correlation. *J. Pharmacol. Toxicol. Methods* 45 227–233. 10.1016/s1056-8719(01)00153-8 11755387

[B32] Piao C. S. Gonzalez-Toledo M. E. Xue Y. Q. Duan W. M. Terao S. Granger D. N. (2009). The role of stem cell factor and granulocyte-colony stimulating factor in brain repair during chronic stroke. *J. Cereb. Blood Flow Metab.* 29 759–770. 10.1038/jcbfm.2008.168 19209180

[B33] Pietrobon D. Moskowitz M. A. (2014). Chaos and commotion in the wake of cortical spreading depression and spreading depolarizations. *Nat. Rev. Neurosci.* 15 379–393. 10.1038/nrn3770 24857965

[B34] Rumbaut R. E. Thiagarajan P. (2010). *Platelet-Vessel Wall Interactions in Hemostasis and Thrombosis.* San Rafael (CA): Morgan & Claypool Life Sciences 21452436

[B35] Saver J. L. (2006). Time is brain–quantified. *Stroke* 37 263–266. 10.1161/01.STR.0000196957.55928.ab 16339467

[B36] Schwamm L. H. Koroshetz W. J. Sorensen A. G. Wang B. Copen W. A. Budzik R. (1998). Time course of lesion development in patients with acute stroke: serial diffusion- and hemodynamic-weighted magnetic resonance imaging. *Stroke* 29 2268–2276. 10.1161/01.str.29.11.2268 9804633

[B37] Sears S. M. S. Hewett J. A. Hewett S. J. (2019). Decreased epileptogenesis in mice lacking the System xc (-) transporter occurs in association with a reduction in AMPA receptor subunit GluA1. *Epilepsia. Open* 4 133–143. 10.1002/epi4.12307 30868123 PMC6398109

[B38] Shih A. Y. Erb H. Sun X. Toda S. Kalivas P. W. Murphy T. H. (2006). Cystine/Glutamate Exchange Modulates Glutathione Supply for Neuroprotection from Oxidative Stress and Cell Proliferation. *J. Neurosci.* 26 10514–10523. 10.1523/jneurosci.3178-06.2006 17035536 PMC6674710

[B39] Shih A. Y. Johnson D. A. Wong G. Kraft A. D. Jiang L. Erb H. (2003). Coordinate regulation of glutathione biosynthesis and release by Nrf2-expressing glia potently protects neurons from oxidative stress. *J. Neurosci*. 23, 3394–3406. 12716947 10.1523/JNEUROSCI.23-08-03394.2003 PMC6742304

[B40] Somjen G. G. (2001). Mechanisms of spreading depression and hypoxic spreading depression-like depolarization. *Physiol. Rev.* 81 1065–1096. 10.1152/physrev.2001.81.3.1065 11427692

[B41] Sommer C. J. (2017). Ischemic stroke: experimental models and reality. *Acta Neuropathol.* 133 245–261. 10.1007/s00401-017-1667-0 28064357 PMC5250659

[B42] Soria F. N. Perez-Samartin A. Martin A. Gona K. B. Llop J. Szczupak B. (2014). Extrasynaptic glutamate release through cystine/glutamate antiporter contributes to ischemic damage. *J. Clin. Invest.* 124 3645–3655. 10.1172/JCI71886 25036707 PMC4109556

[B43] Sosnoski H. M. Sears S. M. S. He Y. Frare C. Hewett S. J. (2020). Sexually dimorphic and brain region-specific transporter adaptations in system x_c_^–^ null mice. *Neurochem. Int*. 141:104888. 10.1016/j.neuint.2020.104888 33199267 PMC7704737

[B44] Swank R. T. Reddington M. Novak E. K. (1996). Inherited prolonged bleeding time and platelet storage pool deficiency in the subtle gray (sut) mouse. *Lab. Anim. Sci.* 46 56–60. 8699821

[B45] Van Liefferinge J. Bentea E. Demuyser T. Albertini G. Follin-Arbelet V. Holmseth S. (2016). Comparative analysis of antibodies to xCT (Slc7a11): forewarned is forearmed. *J. Comp. Neurol.* 524 1015–1032. 10.1002/cne.23889 26494316

[B46] Watson B. D. Dietrich W. D. Busto R. Wachtel M. S. Ginsberg M. D. (1985). Induction of reproducible brain infarction by photochemically initiated thrombosis. *Ann. Neurol.* 17 497–504. 10.1002/ana.410170513 4004172

[B47] Wolfer D. P. Crusio W. E. Lipp H. P. (2002). Knockout mice: simple solutions to the problems of genetic background and flanking genes. *Trends Neurosci.* 25 336–340. 10.1016/s0166-2236(02)02192-6 12079755

[B48] Wolfer D. P. Lipp H. P. (2000). Dissecting the behaviour of transgenic mice: is it the mutation, the genetic background, or the environment? *Exp. Physiol.* 85 627–634. 10.1111/j.1469-445x.2000.02095.x 11187958

