# Supplementary material for: The Cystine/Glutamate Antiporter, System xc–, Contributes to Cortical Infarction After Moderate but Not Severe Focal Cerebral Ischemia in Mice
Source: Front Cell Neurosci. 2022 May 9;16:821036. doi: 10.3389/fncel.2022.821036 (PMC9165760; doi:10.3389/fncel.2022.821036)
Supplement: Supplementary file 1 [file Data_Sheet_1.pdf]

S1. Schema Depicting Timeline of Ischemic Stroke Protocols. Schema depicting the photothrombosis-induced (PTI) ischemia (A, B) and permanent middle cerebral artery occlusion protocols. Detailed descriptions can be found in materials and methods.

S2. Measurement of Cerebral Blood Flow using Laser Scanning Contrast Imaging: Representative single exposure laser speckle contrast imagery of C3H/HeSnJ mouse before and after photochemical induction of stroke (PTI) as described in materials and methods and schematically depicted in S1. Upper panel: real-time tissue blood perfusion graph before and immediately after laser illumination (PTI). Lower panels show region of interest/illumination (ROI), which is encircled. Left panel: LSCI of ROI before and after illumination. Middle panel: bright field image of the chosen ROI. Right panel: depicts the mouse's position and location of the ROI. Video was exported as 4x speed of original recording.
