# Supplementary figures and images for: The Cystine/Glutamate Antiporter, System xc–, Contributes to Cortical Infarction After Moderate but Not Severe Focal Cerebral Ischemia in Mice
Source: Front Cell Neurosci. 2022 May 9;16:821036. doi: 10.3389/fncel.2022.821036 (PMC9165760; doi:10.3389/fncel.2022.821036)

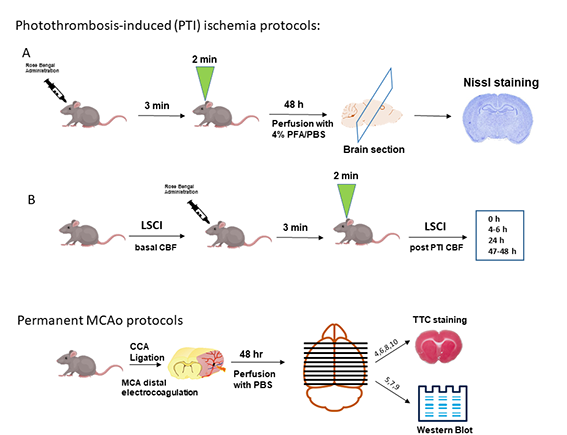

Supplement: Supplementary file 2 [file Image_1.TIF]
